# Comparison of markers of oxidative stress, inflammation and arterial stiffness between incident hemodialysis and peritoneal dialysis patients – an observational study

**DOI:** 10.1186/1471-2369-10-8

**Published:** 2009-03-12

**Authors:** Robert G Fassett, Ritza Driver, Helen Healy, Dwarakanathan Ranganathan, Sharad Ratanjee, Iain K Robertson, Dominic P Geraghty, James E Sharman, Jeff S Coombes

**Affiliations:** 1 Renal Research, Royal Brisbane and Women's Hospital, Brisbane, Queensland, 4029, Australia; 2 Renal Research Tasmania, Launceston General Hospital, Launceston, Tasmania, 7250, Australia; 3 School of Human Life Sciences, University of Tasmania, Launceston, Tasmania, 7250, Australia; 4 School of Human Movement Studies, The University of Queensland, St Lucia, Queensland, 4072, Australia; 5 School of Medicine, The University of Queensland, Queensland, 4072, Australia

## Abstract

**Background:**

Patients on peritoneal and hemodialysis have accelerated atherosclerosis associated with an increase in cardiovascular morbidity and mortality. The atherosclerosis is associated with increased arterial stiffness, endothelial dysfunction and elevated oxidative stress and inflammation. The aims of this study are to investigate the effects of peritoneal and hemodialysis on arterial stiffness, vascular function, myocardial structure and function, oxidative stress and inflammation in incident patients with end stage kidney disease.

**Methods:**

This is an observational study. Eighty stage five CKD patients will be enrolled and followed for one-year. Primary outcome measures will be changes in 1) arterial stiffness measured by aortic pulse wave velocity, 2) oxidative stress assessed by plasma F_2 _isoprostanes and 3) inflammation measured by plasma pentraxin-3. Secondary outcomes will include additional measures of oxidative stress and inflammation, changes in vascular function assessed using the brachial artery reactivity technique, carotid artery intimal medial thickness, augmentation index and trans thoracic echocardiography to assess left ventricular geometry, and systolic and diastolic function. Patients will undergo these measures at baseline (6–8 weeks prior to starting dialysis therapy), then at six and 12 months after starting dialysis.

**Discussion:**

The results of this study may guide the choice of dialysis modality in the first year of treatment. It may also lead to a larger study prospectively assessing the effect of dialysis modality on cardiovascular morbidity and mortality.

**Trial Registration:**

ACTRN12609000049279

## Background

End stage kidney disease (ESKD) patients undergoing dialysis have a substantially increased risk of cardiovascular morbidity and mortality[1] In 2007, 2311 new patients started dialysis in Australia and New Zealand but, unfortunately, many of these patients will die of cardiovascular disease. (ANZDATA 2007) The decision to undergo either peritoneal or haemodialysis is based on a number of factors. One of the important issues is the potential damage the renal replacement therapy may have on the cardiovascular system. Currently, little is known regarding the differential effects of the dialysis modalities on the cardiovascular system.

Oxidative stress and inflammation are associated with the development of cardiovascular disease in dialysis patients [1,2]. The mechanisms are believed to involve arterial stiffening leading to impaired vascular function [3]. In addition, vascular and myocardial dysfunction are also prominent, increasing the likelihood of cardiovascular disease morbidity and mortality in dialysis patients [4]. A number of studies have documented that peritoneal dialysis is associated with decreased levels of oxidative stress and inflammatory markers compared to haemodialysis [5]. A small number of trials have extended this work to determine associations between oxidative stress and inflammation with vascular or myocardial structure and function with equivocal results [4,6]. To our knowledge, only a few cross sectional studies have compared the effects of different dialysis therapies on surrogate markers of cardiovascular disease [7,8]. As yet, no study has compared the changes in these markers over time in incident dialysis patients.

Measures of arterial stiffness such as aortic pulse wave velocity (PWV) predict morbidity and mortality in patients with kidney disease [9]. Numerous studies have reported that the elevated levels of oxidative stress and inflammation in this population [10-14] are associated with arterial stiffness [15]. Therefore, the purpose of this study is to assess the effect of peritoneal and hemodialysis on arterial stiffness, vascular function, oxidative stress and inflammation and myocardial structure and function. Comparing the effects of dialysis modalities on surrogate cardiovascular markers over time will provide unique important information to assist in the treatment of patients with ESKD. A randomised controlled trial assessing this would be ideal but unethical in Australia as patients are generally given a choice between hemodialysis and peritoneal dialysis.

## Methods

### General Considerations and Study Population

This is a multi-centre, one-year prospective observational study utilising convenience sampling. Eighty stage five CKD patients, not yet on dialysis, will be enrolled. They will commence either haemodialysis or peritoneal dialysis according to clinical need and personal preference after undertaking pre-dialysis education. They will be followed for one year after the commencement of their treatment modality. Although this design may be a limitation it is the only ethical one for our aims.

### Location

Patients will be recruited from the Royal Brisbane and Women's Hospital, Brisbane, and the Launceston General Hospital and Satellite Renal Unit Burnie which service populations of approximately 1.2 million and 250,000 respectively.

### Inclusion Criteria

Patients must satisfy the following criteria prior to entering the study: 1) age > 18 and < 85 years, 2) an estimated glomerular filtration rate (eGFR) of < 15 mL/min/1.73 m^2^, 3) undertaken pre-dialysis education program, 4) provide written informed consent.

### Exclusion Criteria

Failure to gain consent or inability to tolerate glyceryl trinitrate (GTN).

### Allocation

The trial is utilising a sample of convenience recruiting participants whom will likely commence either haemodialysis or peritoneal dialysis within three-six months. Approximately six-eight weeks prior to commencing their treatment modality, all eligible patients will undergo baseline measurements of vascular and myocardial structure and function and blood will be collected for the oxidative stress and inflammatory measures. They will then undergo the same measurements at six and 12 months after commencing their chosen treatment.

### Selection and Recruitment of Patients

The nephrologists, trial co-ordinators and research assistants will be responsible for the selection of all eligible patients to be approached. Medical records will be screened prior to attendance at pre-dialysis education clinics. At this stage patient preference for dialysis treatment modality is discussed. After explanation of the study, the subject will be provided with a patient information sheet and informed consent form. The subject will then be asked to take this away with them and arrangements will be made to follow up via a telephone call. If the subject agrees to participate, they will sign the consent form.

### Blood Sampling

A trained phlebotomist will take 40 millilitres of blood during visits to their hospital pathology laboratory or taken on dialysis once this has started.

### Ethical Considerations

The Tasmanian Statewide Scientific and Ethics Committee has approved the study. The Ethics Committee will be provided with annual reports of the trial progress and will promptly receive all adverse event reports.

### Primary Objectives and Primary Outcome Measures

The primary objectives are to assess the effects of dialysis modality on arterial stiffness and oxidative stress and inflammation. The hypothesis is that peritoneal dialysis will significantly improve the rates of change in these measures compared with hemodialysis. The primary outcome measures are outlined below.

#### Arterial stiffness

The primary outcome for arterial stiffness will be aortic PWV. Carotid to femoral PWV will be derived by electrocardiography-gated sequential applanation tonometry (SPT-301 Mikro-Tip, Millar Instruments, Houston, Texas) using the foot-to-foot method (SphymoCor™ 7.01 AtCor, Sydney, Australia) [16]. Testing is performed in a temperature-controlled room and after 5–10 minutes rest with the subject in the supine position prior to the first measure. Two measures will be averaged for the estimation of aortic PWV.

#### Oxidative stress and inflammation

The primary outcome measure for oxidative stress will plasma F_2 _isoprostane measured using gas chromatography mass spectrometry [17] and for inflammation, pentraxin 3 using an ELISA assay [18].

### Secondary Objectives and Secondary Outcome Measures

Secondary objectives are to assess the effects of dialysis modality on vascular structure and function, myocardial structure and function and additional measures of arterial stiffness, oxidative stress and inflammation.

#### Vascular Structure and Function

Carotid Intimal Media Thickness (CIMT) will be used to measure structural alterations of the posterior wall of both the left and right common carotid arteries. CIMT refers to the combined thickness of the intimal and medial layers of the carotid arterial wall [19]. This corresponds to the inner and outer echogenic lines seen on the B-mode ultrasound image [20]. The CMIT will be assessed using a 12 MHz linear array transducer and ultrasound scanner (Vivid i, GE Healthcare, USA). Images will be acquired from the anterior, posterior and lateral planes of the right and left common carotid arteries. The thickness will be measured one to two centimetres proximal to the bifurcation and during cardiac diastole from the far wall only as there are technical and acoustic difficulties encountered when measuring the near wall thickness [20]. For the purpose of this study, the average of these six recordings will be used. CIMT assessment will be performed in the plaque-free arterial wall (an atherosclerotic plaque being defined as an echo-structure protruding into the vascular lumen and a thickness greater by at least 50% than neighbouring sites [21].

Brachial artery reactivity (BAR) will be measured to assess vascular function. A 12 MHz linear array transducer and ultrasound scanner (Vivid i, GE Healthcare, USA) will be used to record the dilator response of the brachial artery to increased blood flow generated during reactive hyperaemia of the downstream forearm. The measures will be performed on the non-fistula arm (if a fistula is present). A blood pressure cuff is placed on the upper aspect for the lower forearm. The brachial artery will be scanned in the longitudinal plane, acquiring a baseline image about five cm above the antecubital fossa. The transducer is left in this scanning position. The cuff is then inflated to 220 mmHg for four minutes. Following cuff deflation, images will be acquired at five and 10 seconds then at 10-second intervals up to 120 seconds. Following a 15-minute rest, this process is then repeated. The 15-minute interval allows the artery to return to its baseline state. The last phase of the BAR test involves the administration of sublingual GTN. Following a final 15-minute interval, a baseline image is acquired and then 300 μg of sublingual GTN will be administered. After three minutes, images of the brachial artery will be obtained as described above. All images will be stored for later offline analysis and archiving. The truest diameter of the brachial artery is indicated by the "double line sign", which is the crisp intima-media and media-adventitia boundaries on the near and far walls [22]. For all measures, the average of three diameter measurements made along the artery, from the trailing edge of intima-media to the leading edge of the media-adventitia, as described by Aeschlimann et al [22], will be used. BAR will be expressed as maximal dilatation (mm), and expressed as a percentage change from baseline. The area under the diameter/time curve (AUC) will also be used to determine temporal changes using analytical imaging software (Imaging Research, St. Catherine's, Canada) and confirming with trapezoidal integration in Microsoft Excel (Seattle, WA, USA) as we have done previously [23]. These will then be compared with the dilatation in response to the administration of GTN. This provides a ratio of endothelial dependant and independent vascular function.

#### Myocardial Structure and Function

Transthoracic echocardiography (TTE) will be performed to assess left atrial and left ventricular (LV) geometry and myocardial systolic and diastolic function. The Vivid *i *(GE healthcare, USA) with a phased-array ultrasound probe (S3) will be used for these measures. Patients will be positioned in the left lateral recumbent position.

LV dimensions will be obtained using two-dimensional (2D) and M-Mode measurements from the parasternal long axis window. Measurements of the interventricular septal wall thickness during diastole (IVSTd), posterior wall thickness (PWT), LV internal dimension during diastole (LVIDd) and LV internal dimension during systole (LVIDs) will be measured to derive the Left Ventricular Mass (LVmass).

LV mass will be calculated from LV linear dimensions using the ASE recommended formula:

LVmass = 1.04 ((LVIDd + PWT + IVSTd (3 - LVIDd)^3 ^× 0.8 + 0.6 g

These measures will then be divided by body surface area to obtain Left Ventricular Mass Index (LVMI).

Relative wall thickness (RWT) will be calculated using the American Society of Echocardiography (ASE) recommended formula [24]:

(2× PWTd)/LVIDd

Patients will have concentric hypertrophy if RWT > 0.42 with increased LV mass; eccentric hypertrophy if RWT < 0.42 with increased LV mass and concentric remodelling if the LV mass normal and RWT was increased[24]. Left atrial (LA) volume will be measured then indexed to BSA, using the area-length (L) method from the apical 4 (A4C) and apical 2 (A2C) chamber views in accordance with ASE guidelines [25]. L will be measured from the mid mitral annular plane and mid posterior LA wall, along its true long axis at end systole. During planimetry, the confluences of pulmonary veins and LA appendage are excluded.

LA Volume = (A_1_)(A_2_)/L × 0.85/BSA[26]

Where L = shorter length from either A4C or A2C. A_1 _= Maximal LA area from A4C. A_2 _= Maximal LA area from A2C.

LV systolic function will be assessed by calculating the ejection fraction (EF) using the disc summation method (Modified biplane Simpson's rule) [25]. Applying the following formula derives EF%:

$$\text{EF}\%=\frac{\text{LVEDV}-\text{LVESV}}{\text{LVEDV}}\times 100$$

Where LVEDV = left ventricular end diastolic volume, LVESV = left ventricular end systolic volume. For the purpose of this study, a normal EF will be ≥ 55% [25].

LV Diastolic Function will be performed using pulsed Doppler interrogations of the LV inflow tract from the A4C view. This will allow for the measurement of peak flow velocity of early (E) and late (A) LV filling. Early diastolic flow (deceleration time DT) and mitral A wave duration. The isovolumic relaxation time (IVRT) will be recorded from the A5C view using continuous wave Doppler interrogation.

Tissue Doppler Imaging (TDI) will measure the longitudinal velocities within the myocardium from the apical 4-chamber view, in pulse wave (PW) mode. This normally consists of a positive systolic wave (S') and 2 diastolic peaks, one during early diastole (E') and the second during atrial contraction (A'). The mitral E velocity and annular E' will be compared as a ratio (E/E'). An E/E' > 10 from the lateral wall is will be indicative of elevated LV filling pressures [26]. Recordings will be made from the lateral mitral annulus.

Pulse wave analysis (PWA) will be used to derive a central (ascending aortic) pressure waveform via applanation tonometry. This method involves using a generalised transfer function to generate a central pressure waveform, which we have shown to be valid [27]. Is a reproducible technique for measuring central blood pressure during hemodynamic perturbations induced by exercise [28]. The central waveform will be calibrated by the average of two measures of brachial blood pressure using a semi-automated device (UA-767, A&D). Central pulse pressure, central systolic blood pressure and augmented pressure will be recorded as markers of LV afterload.

#### Additional Measures of Arterial Stiffness

Augmentation index (AIx) is regarded as a measure of systemic arterial stiffness that will be derived from the central and radial pressure waveforms as previously described [29]. The ratio of brachial to central pulse pressure (pulse pressure amplification) will also be measured as a surrogate of large artery stiffness [30].

#### Oxidative Stress and Inflammation

Additional secondary outcome measures for oxidative stress and antioxidant status will include plasma protein carbonyls, total antioxidant capacity, plasma antioxidant enzyme activities, and for inflammation, C reactive protein and a cytokine panel consisting of IL-6, IL-8, IL-10 and TNF-alpha.

Additional data to be collected will include self-reported health (SF-36 questionnaire), physical activity levels (items from the Active Australia questionnaire) nutritional status (four-day diet diary), adverse events including mortality, morbidity, hospitalizations and procedures performed within the day procedure unit (e.g. iron infusions and arterio-venous fistula formation).

### Visit One (baseline data)

**Figure 1:**
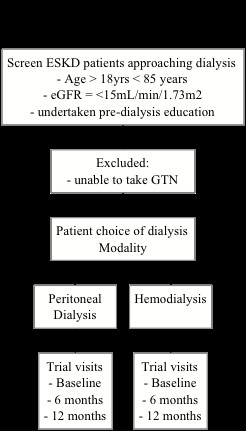
**Flow chart of the study**.

The study flow is summarized in Figure 1 and the study evaluations are outlined in Table 1. After obtaining informed consent, patients will be contacted by telephone by the trial coordinator at which a date and time is agreed for the subject to attend an appointment for baseline trial measures. A letter of confirmation of this appointment and a pathology request form will be posted (if consent formed is signed). Patients will be asked to attend the pathology laboratory at least seven days before their first trial visit to have a fasting blood sample collected. At the first trial visit, if not already done so, the consent form will be signed and additional data will be obtained from the medical records (medical history, medications) and measures of height, weight and blood pressure will be recorded. PWV, PWA, CIMT, TTE and BAR measures will then be made. At the completion of baseline data acquisition, the trial coordinator provides the subject with a pathology request form, for which blood samples are taken within the first week of visit one. The subject will be provided with two questionnaires (SF 36 and items from Active Australia) and a four-day diet diary for completion to take home. The subject is supplied with a self-addressed express post envelope. Subjects are asked to complete and post these within the following two weeks. Data obtained are transcribed onto case record forms for entry into a specifically designed trial database. Subjects are then reminded that they will be contacted by telephone in approximately five months to confirm an appointment date and time for visit two.

**Table 1:** Outcome measures at baseline, 6 and 12 months

Primary Outcome Measures	
Arterial Stiffness	– Aortic Pulse Wave Velocity (PWV)
Oxidative Stress	– Plasma isoprostanes
Inflammation	– Plasma pentraxin 3
Secondary Outcome Measures	
Vascular Structure	– Carotid artery Intimal Medial Thickness (CIMT)
Vascular Function	– Brachial Artery Reactivity (BAR)
Myocardial Structure	- Left Ventricular Mass (LVmass)
	- Left Ventricular Mass Index (LVMI)
	- Left Atrial Volume
	- Left Ventricular Geometry
Myocardial Function	- Ejection Fraction (EF)
	- Early diastolic flow deceleration time (DT)
	- Isovolumic relaxation time (IVRT)
	- Ratio of mitral E and lateral E' (E/E')
	- Left ventricular afterload (central pulse pressure, central systolic pressure, augmented pressure)
Arterial Stiffness	- Augmentation Index (AIx)
	- Pulse pressure amplification
Oxidative Stress	– Plasma protein carbonyls
Antioxidant status	- Plasma total antioxidant capacity
	- Plasma antioxidant enzyme activities
Inflammation	– C reactive protein, IL-6, IL-8, IL-10 and TNF-alpha
Additional blood measures	- Full blood profile, electrolytes, liver function
	- Lipid profile
Self reported health	– SF 36
Physical Activity	– Items from the Active Australia questionnaire
Nutrition	– Four day diet diary
Adverse events	

### Visits Two and Three

These visits occur at six months and one year after commencement of dialysis. An envelope containing a letter confirming appointment, pathology request forms, four-day diet diary, SF 36 and physical activity surveys will be mailed to patients. They will be asked to complete surveys and the diet diary and to attend the pathology laboratory seven days prior to visits. At these visits, data acquisition from measures and tests described above will be performed again. Patients will be examined on a non-haemodialysis day for hemodialysis patients and during the non-dwell phase period for peritoneal dialysis patients.

### Withdrawal from Study

Subjects will be withdrawn from the study at their request, without prejudice, as documented and explained at the time of consenting.

#### Sample Size Estimates

Data from other studies indicates that pentraxin-3 has the highest variability of the three primary outcome measures (mean ± SD; isoprostanes = 54.1 ± 7.0 [46], PWV = 11.4 ± 2.4 [47], pentraxin 3 = 0.75 ± 0.3 [48]) and therefore will be used to estimate the sample size required. We assume that a decrease of 10% in pentraxin-3 would be clinically significant. Therefore, with alpha = 0.05 and beta = (1–0.8), we would need 23 patients in one of the groups. It is expected with an initial enrolment of 80 patients, 60 will complete the one-year measures. Based on the current distribution patterns within the renal services we estimate that 37 of these patients will be receiving haemodialysis and 23 receiving peritoneal dialysis.

#### Statistical analysis

All baseline continuous variables between groups will be compared using general linear modelling, whilst categorical variables will be compared using exact logistic regression. The rates of change will be estimated for each patient separately by linear regression. general linear modeling, will be used to compare the mean rate of change in primary outcome measures between groups, unadjusted and adjusted for actual and potential confounding variables. Mean differences, 95% confidence intervals and P-values will be corrected for repeated measures, and P-values corrected for multiple comparisons by the Holm method. All analyses will be performed using Stata/IC 10.1 for Windows (StataCorp LP, College Station, Tx).

## Discussion

The objective of this study is to assess whether peritoneal dialysis will have less effect on arterial stiffness, vascular function, myocardial structure and function, oxidative stress and inflammation than hemodialysis. Comparing the effects of renal replacement therapy modalities on surrogate cardiovascular markers over time will provide unique important information to assist in the treatment of patients with ESKD. Data from this trial may also lead to a larger scale study assessing the effect of dialysis modality on major cardiovascular endpoints.

## Competing interests

The authors have no competing interests to declare. Baxter Healthcare, although providing a proportion of funding for the study through a competitive grant process, has no influence on the outcome. Although Baxter Healthcare is a provider of peritoneal dialysis therapy it is not the sole provider to the Hospitals included in this study. The outcome of the study will not impact on the balance of product use after completion of the study. However, the study outcome may influence the distribution of the use of peritoneal dialysis compared with hemodialysis in the first twelve months of therapy. This could result in an increased or decreased use of either dialysis therapy or in fact no alteration.

## Authors' contributions

RGF and JSC are responsible for the design of this clinical trial, the construction of the protocol and writing this manuscript. HH, DR and SR provided protocol advice and will be involved with recruitment and retention, manuscript review and data analysis. IKR has and will provide statistical advice. DPG provided technical advice and manuscript review. RD will perform measures of vascular and cardiac structure and function and provided protocol design elements related to these. JES provided advice on vascular measures and manuscript review. All authors read and approved the final manuscript.

## Pre-publication history

The pre-publication history for this paper can be accessed here:

http://www.biomedcentral.com/1471-2369/10/8/prepub
